# Bone Regeneration Induced by Strontium Folate Loaded Biohybrid Scaffolds

**DOI:** 10.3390/molecules24091660

**Published:** 2019-04-27

**Authors:** Marcela Martín-del-Campo, José G. Sampedro, María Lisseth Flores-Cedillo, Raul Rosales-Ibañez, Luis Rojo

**Affiliations:** 1 Departamento de Biomateriales, Instituto de Ciencia y Tecnología de Polímeros, CSIC, 28006 Madrid, Spain; 2 Instituto de Física, Universidad Autónoma de San Luis Potosí, Manuel Nava 6, Zona Universitaria, San Luis Potosí C.P. 78290, S.L.P., Mexico; sampedro@dec1.ifisica.uaslp.mx; 3 División de Ingeniería Industrial, Instituto Tecnológico Superior de San Luis Potosí, Capital, Carretera 57 Tramo Qro-SLP Km 189+100 No. 6501, Deleg, Villa de Pozos, San Luis Potosí C.P. 78421, S.L.P., Mexico; maria.flores@tecsuperiorslp.edu.mx; 4 Escuela de Etudios Superiores, Iztacala, Universidad Nacional Autónoma de Mexico, UNAM, Tlalnepantla 54090, Mexico; rosales_ibanez@unam.mx; 5 Consorcio Centro de Investigación Biomedica en red, CIBER-BBN, 28029 Madrid, Spain

**Keywords:** bone regeneration, musculoskeletal defects, strontium folate

## Abstract

Nowadays, regenerative medicine has paid special attention to research (in vitro and in vivo) related to bone regeneration, specifically in the treatment of bone fractures or skeletal defects, which is rising worldwide and is continually demanding new developments in the use of stem cells, growth factors, membranes and scaffolds based on novel nanomaterials, and their applications in patients by using advanced tools from molecular biology and tissue engineering. Strontium (Sr) is an element that has been investigated in recent years for its participation in the process of remodeling and bone formation. Based on these antecedents, this is a review about the Strontium Folate (SrFO), a recently developed non-protein based bone-promoting agent with interest in medical and pharmaceutical fields due to its improved features in comparison to current therapies for bone diseases.

## 1. Introduction

The rise in the average age of the population has led to a steady increase in the number of musculoskeletal conditions and in particular of cartilage and bone surgical procedures in the last years [1,2]. Therefore, the development of alternative materials and strategies in bone replacement therapies has attracted high interest. Above a critical size, bone defects are not repaired by the self-healing system of the tissue; thus, an osteoconductive and osteoinductive device (or scaffold) is usually required in order to regenerate the lost tissue. The scaffold must be composed of materials that stimulate and favor the formation of new bone tissue as well as to be structurally stable during the process of cell growth and expansion [3]. In this regard, autografts are still amply considered as the “gold standard”; however, they have many drawbacks such as limited availability and morbidity of the donor site [4,5]. Alternatively, a proper scaffold made of a given biomaterial would be desirable both to fill the defect and to act as a reservoir for growth factors and/or cells [6,7].

High biocompatibility and proper physical-chemistry of the surface are the most essential requirements in biomaterials intended to be used in scaffold construction [6,7]. Nonetheless, several others important criteria must also be taken into account; these are cell adhesion, proliferation and differentiation, biodegradability, mechanical efficiency (in a given specific application), bio-conductivity, bioactivity, suitability to the sterilizing procedure, stability during storage, and finally, high cost-effectiveness ratio [6,7]. Polymers are usually good candidates despite only few are those displaying most of the characteristics required for their use as bone substitutes [8]. Additionally, some polymers may generate inflammation (an immunological reaction to foreign body). Such reaction usually leads to the failure of the engineered product, and thus severely restricting the use of these polymers as scaffolds [9]. To date, unfortunately, there are no materials with the desired characteristics to construct an optimal scaffold, i.e., with the properties required to generate new bone tissue [10]. Currently, the employment of different types of technologies as well as the generation of new polymeric synthetic scaffolds has become an attractive option in order to overcome the drawbacks of synthetic and natural polymers [11]. Furthermore, the inclusion of metal ions and trace elements essential in bone formation may be considered in scaffold synthesis, e.g., calcium (Ca^2+^), magnesium (Mg^2+^), and phosphate ions (PO_4_^3−^), while metallic trace elements when incorporated into bone structure promote both osteogenesis and angiogenesis thus enhancing bone remodeling and the repair process [12]. Notably, amongst the metallic trace elements, Sr has been extensively studied [10]. Sr, because of its resemblance (in charge and size) to calcium (Ca), has the ability to substitute Ca ions in the apatite structure [10,13]. Jimenez et al. reported that Sr induces human stem cell differentiation towards cartilage and bone like phenotypes in vitro. Thus, biomaterials containing Sr would enhanced their performance both in vitro and in vivo, that is, by inducing the formation of new bone and cartilage tissues while inhibiting tissue resorption [14].

Certainly, Sr stimulates the activity of osteoblasts while inhibiting the activity of osteoclasts [14]. In this regard, Place et al. evaluated the osteogenic potential and showed that Sr may improve osteogenic differentiation [15]. Accordingly, some authors performed the transplantation of strontium-based systems in bone defects [16,17]; computed tomography and histological analysis showed a significant improvement in bone formation in vivo, i.e., the amount of both mature and remodeled bone substantially increased and extracellular matrix accumulated [16,17]. Further studies showed that Sr increased the expression of β-catenin in the newly formed bone, and such an effect was expected to occur either in vitro and in vivo, e.g., a significant osteogenic differentiation of mesenchymal stem cells (MSCs) was expected when in the presence of Sr, and consequently, the formation of bone [15,16,17,18]. Additional studies suggested the incorporation of Sr ions in bone substitutes but were also shown to be effective in the stimulation of the proliferation and differentiation of bone MSCs, osteoblasts, and periodontal ligament cells in vivo [19,20,21]. Hence, a three-dimensional (3D) biohybrid scaffold based on calcium phosphate and a bio-active derivative of Vitamin-B and Sr (Strontium Folate, SrFO) was developed. This device induced the healing of critical-sized cranial defects in rats using human dental pulp stem cells (HDPSCs) without the addition of exogenous growth factors. The seeded HDPSCs proliferated and differentiated, hence developing a bone regenerative capacity. This seems to be a novel tissue engineering approach to regenerate critical sized bone defects in vivo [16]. Due to the high potential of SrFO in bone regenerative medicine, currently, new regenerative devices have been developed based on SrFO. Very recently, Xu et al. demonstrated that strontium folic acid derivatives promote in vitro the osteogenic differentiation of MSCs shortly after addition; especially in the long term. Particularly, such derivatives improved significantly bone formation around the bio-functional orthopedic implants in vivo, which was highly evident at late stages [17].

## 2. Factors Involved in the Repair of Bone Tissue

The process of bone tissue repair involves a complex cascade of biological events controlled by numerous cytokines and growth factors (GF). These factors provide the signals that induce the migration of osteoprogenitor cells and promote the subsequent differentiation and proliferation in cell lineages, tissue revascularization, and production of the extracellular matrix [22]. Therefore, the inclusion of growth factors, as well as bone morphogenetic proteins (BMPs), and fibroblastic growth factors (FGFs) in scaffolds seeded with stem cells have been demonstrated to be important in the effectiveness of the regenerative process [23]. However the use of these GF in clinical applications has been restricted due to their limited availability in terms of price, shelf life during storage and manipulation, and the short time in effectiveness after their application due to hydrolysis, neutralization, and degradation.

Bone tissue consists mainly of the protein collagen (~90%), and of cells with a role in bone formation and growth, repair and synthesis of the extra cellular matrix [23,24]. The matrix is formed by a small binding integrin-linked to glycoprotein N. The sibiling protein family is formed by five members: (a) osteopontin (OPN), (b) fosfoglycoprotein of the extracellular matrix (MEPE), (c) bone sialoprotein (BSP), (d) dentin matrix protein 1 (DMP1), and (e) dentin sialophosphoroprotein (DSPP). The members of the family share some structural characteristics, namely: collagen binding domain, HA binding domain, and arginine-glycine-aspartic acid (RGD) cell binding domain [23]. Undoubtedly, these proteins have an important role in bone development, i.e., facilitating cell adhesion, nucleation, and mineral maturation. Nonetheless, researchers are still trying to elucidate the exact role of each protein in natural bone development [24,25].

Tumor necrosis factor α (TNFα) is one of the most potent osteoclastogenic cytokines. TNFα stimulates bone resorption in vitro and in vivo by increasing the proliferation and differentiation of osteoclast precursors [26,27]. In contrast, OPG can reverse the loss of bone in animal models of sex-steroid insufficiency and glucocorticoid-induced osteoporosis, rheumatoid arthritis, multiple myeloma, and metastatic bone disease [28]. Since OPG directly counteracts all RANKL mediated activities through RANK, therefore the RANKL/OPG ratio is determinant in bone mass and integrity of the skeleton. In contrast, the increased expression of RANKL by tumor cells and tumor-mediated increase of RANKL/OPG ratio in bone microenvironment may be observed in myelomas and in osteolytic metastasis in prostate and breast cancer [27,29,30,31,32,33].

## 3. Dental Pulp Stem Cells and Osteogenic Differentiation

Stem cells are generally defined as clonogenic cells capable of self-renewal; these are non-specialized cells that renew by cell division with the remarkable ability to differentiate to a specific cellular type [34,35,36]. Since stem cells are able to repair or replace damaged tissues, thus leading to recover from illness (or injury), they have been employed successfully. Therefore, the number of patients subjected to tissue transplants, plus patient receptors of stem cell therapy, has increased recently [11].

**Figure 1:**
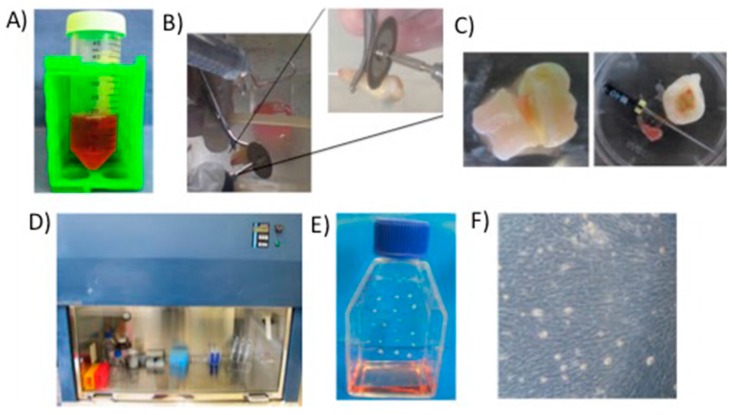
Procedure for culture and proliferation of dental pulp stem cells (DPSCs) used in bone tissue engineering as reported in Martin-del-Campo et al. (**A**) Dental organs stored in culture medium and antibiotics. (**B**) Segmentation of dental organs. (**C**) Extraction of dental pulp. (**D**) Laminar flow hood for cellular handling. (**E**) Generated cellular nodules from cells. (**F**) Expanded Dental Pulp Mesenchymal Stem Cells [16].

Dental pulp has been considered an excellent source of adult stem cells, even better than bone marrow. Hence, research has been focused to test its therapeutic application [3,37], e.g., in tissue engineering, where numerous laboratories have assessed its potential in pre-clinical applications in the regeneration of tissues [33]. Importantly, adult dental pulp tissue does contain multipotential stem cells and under given conditions, the derived pulp stem cell cultures may develop to specialized odontoblasts-like cells competent to form mineralized nodules in vitro [34]. In this regard, dental tissues are easily accessible during routine clinical practice, namely upon tooth extraction; thus providing a source of trustworthy stem cells, ideal for development and testing of new therapies [38]. Technically, stem cells are isolated from pulp tissues degraded from the selection of a unique colony (Figure 1). These stems cells usually exhibit high proliferation and clonogenic properties; they also exhibit the typical immuno-reactivity profile of bone marrow stromal cells [34]. Furthermore, the osteogenic cultures are able to synthesize fibers, similar to bone tissue, thus forming a densely mineralized matrix. These so generated lamellar fibrous bone tissue usually tend to contain osteocytes, after transplantation into living organisms [39].

Studies in engineering bone tissue have demonstrated the potential of DPSCs when in combination with a 3D matrix. Mineralization and hard tissue formation are achieved by partially developed hematopoietic bone tissue [40]. Similar results have been obtained upon implantation of manufactured constructs consisted of DPSCs embedded in scaffolds [16,41]. It is worth to note that this strategy produces a highly mineralized tissue. Thus, generating a deep interest and encouraging the use of DPSCs in clinical bone tissue replacement therapies [41].

## 4. The Use of Strontium in Bone Repair

Strontium is an element that has an approximate volume of distribution of 1l/kg. The Sr binding capacity to human plasma proteins is low (~25%), but display high affinity for bone tissues. Studies showed that Sr elimination is performed by the kidneys and gastrointestinal tract, but independent on time or dose. The effective half-life is ~60 h. Sr plasma clearance is achieved at 12 mL/min (CV 22%), while its renal clearance is achieved at 7 mL/min (CV 28%) [42].

It has been described that Sr is adsorbed on the surface of bone mineral, instead of replacing Ca ions; this favors its rapid elimination. Nonetheless, Sr has a particular ability to cause catabolic and anabolic effects during bone remodeling through the induction of prostaglandin synthesis and expression of cyclooxygenase, which increases the differentiation to osteoblasts from mesenchymal stem cells (MSCs). Peng et al. showed that Sr could increase bone osteoblastic differentiation in vivo, and participate in the process of MSCs differentiation in vitro [43]. However, the mechanism by which Sr affects the signaling pathways leading to osteogenic differentiation in human MSCs resulting in bone formation is unknown [44].

According to in vivo experimental models, Sr is able induce anabolic events promoting bone formation; however, further experimental and clinical analyses on the associated pathways are required to clarify its effects at the molecular level [45]. Furthermore, in a postmenopausal osteoporosis animal model, the long administration of Sr prevented the trabecular loss of bone induced by estrogen deficiency, probably promoting bone formation by increasing bone mineral density and preventing its resorption [45]. In this regard, experimental results showed that Sr administration increases the number of osteoblasts, decreases the number of osteoclasts, and promotes matrix formation. Notably, besides the effect on bone cells population and bone micro-architecture, Sr increases the mechanical resistance of bone through changes in the properties of bone matrix [14,46].

Fernández-Murga et al. performed a global genomic study on the effect of different Sr compounds and a calcium salt in pre-osteoblasts cultures, thus obtaining information on genes and the signaling pathways involved in osteogenesis. Importantly, strontium induces the maturation process of pre-osteoblasts after 21 days of treatment, nonetheless the most notable changes occurs immediately at cellular level on gene expression and activation of signaling pathways related to osteogenic events. Successive waves of changes in gene expression pattern occurs within 0–7 days, which seems to be required to initiate the differentiation of pre-osteoblasts to a mineralizing phenotype. The expression of many genes related to regulation of transcription, metabolic processes and transport of molecules change in this first phase of the maturation process [46]. In this regard, phosphorylation-dephosphorylation of proteins was shown to be the main biochemical event in this initial short time span; phosphorylation-dephosphorylation of proteins is well known to be involved in the regulation of metabolic pathways as well as in other important cellular processes. For instance, Wnt and NFAT signaling pathways were shown to be involved in the maturation of osteoblasts [47,48], as both Wnt/β-catenin and NFAT pathways behaved as potent osteoprogenitors inducing gene expression changes in short times. In contrast, cell cultures treated with strontium showed no activation of these pathways at day 21. Hence, once confluence is reached, that is, when increased ALP activity and the deposit of mineralized matrix are observed, the cells enter into the differentiation phase [46]. This phase is characterized by an increase in the formation of bone matrix, which is associated to the change in expression of 147 genes. In addition, cellular processes related to the energy state of the cell increases remarkably in this second phase, namely carbohydrate metabolism (glucose and other carbohydrates) including gluconeogenesis [46].

Overall, Sr is currently known to stimulate osteoblasts promoting osteogenesis both in vivo and in vitro, while conversely, Sr down-modulates function in osteoclast preventing bone resorption [49,50]. The Sr anabolic effects involve Ca receptors leading to improve: preosteoblast replication, osteoblasts differentiation, synthesis of collagen type I, and mineralization of bone matrix [18,51]. Sr replaces Ca in the metabolism and functions of osteoblasts and also enhances osteogenesis inducing osteoblast division while preventing osteoclast resorption. In addition, Sr enhances osteoblasts differentiation to osteocytes [52]. In this regard, osteocytes modulate the function of both osteoblasts and osteoclasts, thus displaying a role in the uncoupling of bone turnover by producing paracrine signals triggered by the mechanical load [10,53,54,55].

Similarly to Ca, Sr is also absorbed in the intestine and finally distributed between three main compartments, namely in plasma and extracellular fluids, soft tissues, and particularly in bones. Notably, alteration of the Sr/Ca and Sr/P ratios have been linked to skeletal deformities [56]. Pasqualetti et al. stated that bone deformities and other disorders occur at low Ca/Sr ratios, thus resulting in a slower Ca uptake into bone tissue hence facilitating the replacement of Ca by other elements. The Sr usually acts replacing Ca, as it displays physical and chemical similarity to Ca. Sr, once ingested, become present in all body tissues; nonetheless, about 90% of it accumulates in the bones [57]. Sr is slowly excreted by the body, and as suggested by Polak-Juszczak et al. this seems to be the cause of the high Sr and low Ca concentration resulting in low Ca/Sr ratio values [56]. Similarly to Ca, phosphate (PO_4_^3−^) is also essential for proper body development by sustaining the skeletal system. P deficiency results in reduced growth rates and bone mineralization, thus resulting in skeletal anomalies. Importantly, Sr ingestion seems to cause a decreased assimilation of both Ca and P; hence, the values of Ca/Sr and PO_4_^3−^/Sr ratios are in some way related [56].

In several laboratories, the search of materials (as a vehicle for Sr) displaying low cellular toxicity, even better showing synergistic activity in promoting regeneration of bone tissue, is highly active. In this regard, a composite of Sr and Ca polyphosphate (CPP) was described showing excellent mechanical properties with a steady release of Ca, PO_4_^3−^, and Sr ions, which stimulates osteoblasts growth in vitro [58]. In animal models (in vivo), the composite behaves similarly as in vitro, e.g., in the canine femur, Sr-CPP composite promotes considerably bone formation and angiogenesis [59]. Similarly, Li et al. reported a composite consisting of Sr, hydroxyapatite (SrHA), and BisGMA, which showed satisfactory bioactivity [60,61]. Hernandez et al. developed a cement based on SrHA and poly(methyl methacrylate) intended to be used in minimally invasive surgeries, this composite showed excellent bioactivity in vitro and thus, potential use in vivo. Notably, high biocompatibility is observed in the above composites, which is characterized by low cytotoxicity and improved cell proliferation of fibroblast cultures [62].

Therefore, Sr promotes cell proliferation and differentiation, as well as mineralization. Nonetheless, Sr behavior seem to depend to the anion to which it is conjugated [46]; for example, it has been used in combination with ranelate (SrRA) in bone regeneration therapy [63]. The salt has been indicated in postmenopausal osteoporosis treatment showing effects also on cartilage and subchondral bone. Preclinical studies performed indicate that SrRA inhibits subchondral bone resorption and promotes formation of the cartilage matrix in human chondrocytes, both in normal and osteoarthritic conditions [63]. SrRA induces the maturation of osteoblastic cells and increases the synthesis of collagen and non-collagen proteins. Regarding other effects of SrRA in bone formation, reports have shown that it also improves the preosteoblastic cellular replication [64]. However, the use of SrRA is being reviewed currently because of the increased incidence of cardiac failure events and thromboembolism [18], while other important risks have also been reported, namely cutaneous adverse reactions, alterations of consciousness, seizures, hepatitis, and cytopenia (European Medicines Agency, 2011). Therefore, the unfavorable benefit-risk balance for Sr ranelate is clear; as consequence, marketing authorization for pharmaceutical products (Protelos^®^ and Osseor^®^) containing SrRA has been suspended by the Pharmacovigilance Risk Assessment Committee (PRAC) of the European Medicines Agency (EMA). Furthermore, a safety alert on severe allergic reactions has been issued for drugs containing Sr ranelate (European Medicines Agency, 2011). In spite of that, others organic anions are being evaluated in order to improve the bioavailability of Sr and to avoid most of the disadvantages associated with the ranelic moiety [65].

Nonetheless, the development of bone replacement materials has continued to be an important goal in medicine, mainly because of the disadvantages in the use of bone autografts. In this regard, the intended use of Sr to this goal has not vanished and others composites have been tried, e.g., alginate hydrogels prepared with arginine-glycine-modified aspartic acid (RGD) cross-linked with Sr and Zn ions, and including Ca; Zn is required for alkaline phosphatase (ALP) activity. Although Ca and Sr based hydrogels display different stabilities during storage, they have similar stiffness and support the proliferation of osteoblast-like cells. Notably, the release of Sr from alginate gels is constant, sustained, and biologically active. Sr induces positive regulation of phenotypic marker genes in osteoblasts: RUNX2, collagen I (COL1A1) and bone sialoprotein (BSP), while ALP protein activity was maximal in alginate gels containing Sr. The strategy may be extensive to combination with other systems or to adaptation to applications in bone tissue engineering. It has been suggested that hydrogels may be used as a reservoir for the slow-release of Sr leading to improve osteogenic activity and/or differentiation [15]. Remarkably, studies showed that the effectiveness of Sr may be increased when in combination with other ions [15,18].

During osteogenesis, multiple signaling pathways, including Vascular endothelial growth factor (VEGF), runt-related transcription factor 2 (Runx2), osterix (Osx), bone morphogenic protein (BMP), mitogen-activated protein kinase (MAPK), and the wingless-type MMTV integration site (Wnt)/β-catenin pathways, regulate the proliferation and differentiation of osteoblasts, and hence are involved in controlling bone formation. In addition, the increased expression of bone matrix proteins, such as alkaline phosphatase (ALP), type I collagen (ColI), and osteopontin (OPN) also stimulate mineralization and bone formation [66]. Osteoblasts, besides regulates bone formation, also control bone resorption by modulating osteoclastogenesis via osteoprotegerin (OPG)/receptor activator of nuclear factor-κB and ligand (RANKL)/receptor activator of nuclear factor-κB (RANK) system [66,67].

Vascular endothelial growth factor (VEGF) is involved in angiogenesis and vascular homeostasis [68] and plays an essential role for regulating angiogenesis, endothelial cell function, and hence, signaling maturation of osteoblasts, ossification, and bone turnover. [69]. The angiogenic process accompanies bone regeneration acting as a limiting factor for the healing process [70]. Salinas et al. [71] showed a synergistic effect of VEGF adsorbed on silicon substituted hydroxyapatite scaffolds, resulting in more ossification, larger trabeculae and higher degree in angiogenesis [71]. Interestingly, incorporation of Sr in a fluorapatite glass-ceramics system leads to a highly ordered microstructure, increased solubility, and sustained Sr release, demonstrating that the in situ delivery of key elements is an attractive strategy to promote both angiogenesis and osteogenesis [10,72]. The fluorapatite-based glass composite sustained the release of Sr in vitro and in vivo, where the rate of mineral apposition is higher than the Sr undoped composite. Therefore, the efficiency of Sr in promoting osteoblastic activity is clear [10].

Among a series of GFs used in exogenous delivery strategies in bone repair, the bone morphogenetic proteins (such as BMP-2) are well known that promote osteoblastic differentiation of MSCs as well as the regeneration of bone at early phase of formation [73]. BMP-2 in conjunction with biochemical factors (chemical compounds, bioactive cytokines, and drugs) is known to synergistically promote osteogenesis, thus leading to bone regeneration under physiological microenvironment [74]. In example, some vitamins maintain body Ca and PO_4_^3-^ balance, while the drug dexamethasone (Dex) promotes osteogenic differentiation of MSCs in vitro [75,76]. Hence, a synergistic effect of BMP-2 and vitamin D3 in osteogenic differentiation of adipose derived stem cells (ADSCs) has been observed in vitro; a high dose of vitamin D3 upregulates ALP expression and mineralization of ADSCs. Additionally, studies have showed a controlled and sustained release of BMP-2 and Dex using engineered delivery platforms activating bone regenerative processes [73].

BMP-2 is known to induce or promote the expression of RUNX2; a transcription factor essential for osteoblast differentiation and bone formation [77]. Nonetheless, ALP, COL-1, and osteocalcin are also essential in osteoblast differentiation, thus also considered as molecular markers in bone formation [78]. BMP-2 regulates osteoblast differentiation by stimulating osteoblast-related transcription factors, such as RUNX2 and SMAD1, while the closely-related protein, SMAD5 mediates the responses to BMP-2 [73,76]. Bone morphogenetic proteins (BMP) are members of the TGF*β* superfamily. These proteins have diverse functions in multiple developmental processes such as in embryogenesis, organogenesis, bone formation, cell proliferation, and stem cell differentiation [73].

Bone marrow MSC cultures, when exposed to Sr, displays a significant increase in the expression of the master gene, Runx2, and bone sialoprotein (BSP), which are associated to the increase of colony-forming unit osteoblasts. Interestingly, the Sr mediated activation of gene expression varies with differentiation stage of MSC; namely, Runx2 and BSP in bone marrow MSC, Runx2 and osteocalcin in preosteoblasts, and BSP and osteocalcin in mature osteoblasts [55].

## 5. Role of Folic Acid and Vitamins B_6_ and B_12_ in Bone Regeneration

Folate (FO) is a group of chemically complex substances of which the main role is to provide methyl groups in biosynthetic pathways. The human body is unable to synthesize FO; it is obtained from foods, and nowadays also in the form of dietary supplements. FO is essential at all ages in human life, from early development in the uterus to adulthood [79].

Biochemically, FO is a coenzyme that transfers carbon units necessary for the formation of deoxythymidylate used in the synthesis of purine and others methylation reactions. Therefore, folate is essential for cell division, embryonic and fetal development, and in the maintenance of cardiovascular and neurological functions. FO is produced by green plants, especially leafy vegetables, and some microorganisms, and it is found as a derivative of the reduced form of tetrahydrofolate (THF). Ingested FO becomes functional after intestinal absorption, distribution, transport into the cells, and biochemical modification. The lack of nutritional FO supplementation has been related to chronic pathologies such as cardiovascular disease, cancer, and cognitive dysfunction. Importantly, deficient nutritional status in maternal FO (Folic acid and FO derive from the aqueous soluble vitamin B_9_) is related to defects in the neural tube in offspring [79,80]. Therefore, folic acid supplementation during pregnancy is recommended by reproductive health epidemiological studies in different groups of women [81]. Nowadays, the consumption of probiotics is a new alternative to the supplementation of FO, e.g., strains of *Bifidobacterium* producing FO have been tested in humans and animals showing increasing levels of FO in plasma [82].

The effect of folic acid on bone turnover and bone metabolism has been evaluated [83,84]. Published results showed that folic acid supplementation does have beneficial effects on bone health status [85]. The effect of folic acid on bone metabolism and its turnover has been evaluated during pregnancy [27,85]. Results showed that pregnant women with daily folic acid (1 mg) supplementation until the birth time display significant higher plasma levels in OPG concentration and lower in sRANKL concentration than pregnant women with folic acid supplementation up to the second trimester, consequently low rates in bone resorption is implied [85]. Importantly, better results are obtained when higher folic acid dose (5 mg/day) is administered, i.e., the sRANKL/OPG ratio further decreases [27].

Inflammatory cytokines (IL-1, TNF, and M-CSF), which are released when osteoclastic bone is lost, stimulate RANKL production in osteoblast precursors and/or development of osteoblasts [86]. In contrast, these cytokines decrease OPG production through up-regulation of the RANK receptor in osteoclast precursors, thus increasing their sensitivity to normal RANKL concentrations [27,87]. In this regard, studies showed that TNFα in serum decreases significantly during the administration of a high dose of folic acid. The TNFα decrease correlates with the decrease in sRANKL levels. Importantly, TNFα promotes osteoclastogenesis via RANKL system, i.e., by up-regulation of RANKL mRNA expression [88]. Therefore, in pregnant women folic acid supplementation in high dose decrease bone resorptive biomarkers by increasing OPG level and decreasing sRANKL and TNFα levels [27].

B-vitamins are a group of soluble vitamins that are cofactors of some of the enzymes involved in the metabolic pathways of carbohydrates, fats, and proteins; due to their promising properties, researchers have studied the advantages of their use in the clinical field, such as boosting the immune system [89]. B-vitamins are excellent adjuvants to achieve regeneration in several types of tissue. According to Fernández-Villa et al. folic acid derivatives are considered one of the most encouraging derivatives because they fulfill the requirements for their application in clinical use. It has been shown that these derivatives promote regenerative processes in a wide range of tissues and organs; further folic acid derivatives show a long lifespan under physiological conditions. This is the greatest limitation in regenerative therapies; e.g., when using growth factors or other recombinant proteins [90].

B-vitamin has also been linked to osteogenesis. An experimental study from Herman et al. showed a strong stimulation of human osteoclasts by the amino acid homocysteine (HCY) [91]. Other potential effects of HCY-related osteoporosis are reduced osteoblast activity [92] and disturbed extracellular collagen cross-linking [93]. It was proposed that the elevated HCY concentration in vivo is caused mainly by deficiencies in FO, vitamin B12 and B6, as they are directly involved in degradation of HCY. In a large series of experiments using cell cultures, Herrman et al. demonstrated the important role of FO and these B-vitamins in bone metabolism, namely FO, vitamin B12, and vitamin B6 stimulated osteoclast activity by accumulation of HCY [94]. In contrast, in preosteoblastic bone marrow cells, vitamin B12 increases alkaline phosphatase activity in a concentration-dependent way [91].

Likewise, folic acid and FO are important molecules in bone health; vitamin B_9_ derive from both molecules. The most prominent role of vitamin B_9_ is detoxification of HCY related to inflammation and to increased risk of fractures. Studies performed in developed countries showed that women (during menopause) experience a diminished ability to manage homocysteine adequately and that supplementation with folic acid improves the processing of homocysteine [95]. Nonetheless, this is not yet recognized as a universal [96].

Hyperhomocysteinemia seems to have a potential role in the development of osteoporosis. HCY is the aminoacid produced in methionine catabolism. Interestingly, serum concentration of HCY shows an inverse relationship with vitamin B_12_ and folic acid [96]. HCY metabolism is related therefore to methionine concentration, expression of enzymes, concentrations of cofactors (vitamin B_6_ and B_12_), and FO (tetrahydrofolate production). Therefore, a deficiency in enzyme activity or the absence of cofactors may cause accumulation of HCY in cell cytoplasm, but also in blood plasma [97,98]. Importantly, studies have shown an association between high blood plasma HCY levels and osteoporotic fractures in elderly women and men [99]. The proposal that HCY plays a role in bone metabolism is supported by studies examining the relationship between a polymorphism in the methylene tetrahydrofolate reductase gene (MTHFR) and bone mineral density (BMD). However, the exact relationship between the level of HCY in plasma and BMD is uncertain. Methylation of HCY leads to form methionine in a reaction catalyzed by methionine synthase [100]. Methyl-tetrahydrofolate behaves as the donor of the methyl groups using vitamin B_12_ as a cofactor. Methyl-tetrahydrofolate is synthesized from folic acid, in a route where methylenetetrahydrofolate-reductase (MTHFR) participates using FAD (flavin-adenine-dinucleotide) as a cofactor. Therefore, four vitamins (folic acid, vitamin B_6_, vitamin B_12_, and riboflavin) participate in the metabolic reactions leading to decrease HCY levels. Deficiencies in these vitamins increase homocysteinemia, this in turn impact in bone mineral density [100].

A relationship between homocysteine levels and the incidence of fractures seems to exist in individuals [98,101]. However, no relationship has been found between homocysteine levels in plasma and density in bone mineral [101]. Nonetheless, other studies have shown that folic acid and vitamin B_12_ deficiency is specifically associated with a decrease in BMD and a high incidence of fractures [102,103]. Since folic acid, vitamin B_12_, and homocysteine are metabolically related, their specific influence in skeletal homeostasis becomes difficult to determine; however, when analyzed in conjunction, a statistically significant association is observed in the concentration of folic acid and vitamin B_12_ with BMD [100].

BMD and bone mineral content are known to be influenced by Vitamin B_12,_ Ca, and vitamin D. In this regard, studies in vitro show that vitamin B_12_ does have a significant effect on osteoblasts proliferation, e.g., vitamin B_12_ increases alkaline phosphatase activity in osteoclastic cells. A minute amount of vitamin B_12_ is necessary to observe a positive effect on osteoblasts proliferation. In elderly patients, Dhonukshe-Rutten et al. found a relationship between vitamin B_12_, BMD and bone mineral content [104]. Therefore, as vitamin B_12_ seems to be related to bone health, the treatment of vitamin B_12_ deficiency may help to prevent osteoporosis. Additional studies in elderly women showed an association between vitamin B_12_ levels, its content in bone mineral, and BMD [104]. Therefore, the decrease or increase in vitamin B_12_ levels seems to be related to BMD, and thus to the susceptibility or not to bone fractures, respectively. In agreement, the observed prevalence ratio in osteoporosis was higher in groups with deficient to marginal vitamin B_12_ than those with normal levels. Studies suggest that clinical deficiency in vitamin B_12_ may be associated with poor functional maturation of osteoblasts and thus bone formation and resorption, respectively [91]. Vitamin B_12_ participates as a cofactor in the transformation of homocysteine to methionine; however, the mechanism by which bone density is affected is not fully understood [96].

## 6. Synthesis and Properties of SrFO

Currently, the development of new osteogenic therapies is on clinical demand, namely in the treatment of osteoporosis and other bone diseases. The therapy based on Sr seems promising; nonetheless, any formulation should provide an effective and consistent way to deliver Sr ions with low or absence of secondary pharmacological effects. In this regard, Rojo et al. [18] developed a new strategy for Sr delivery based on FO as the carrier [18]. Experimental results showed the improvement in the regenerative capacity of skeletal tissues; thus evidencing a high efficiency of Sr as the active compound. Certainly, FO (the anionic form of folic acid) is a promising alternative as a Sr carrier in composites [18]. Additionally, the incorporation of B vitamins (folates) may contribute improving the metabolism and other processes in cells involved in bone regeneration [90].

**Figure 2:**
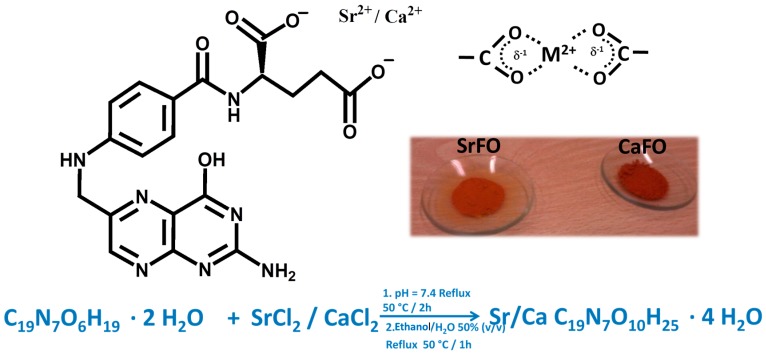
Chemical structure of calcium/strontium folic acid derivatives with bidentate coordination structure and synthetic conditions yielding pure powdered compounds. Figure adapted from Rojo et al. [18] with permission from the Royal society for Chemistry.

Ca and SrFO complexes have been obtained and characterized physicochemically, and their bioactive properties in osteoporosis treatment have been evaluated (Figure 2) [18,58].

**Figure 3:**
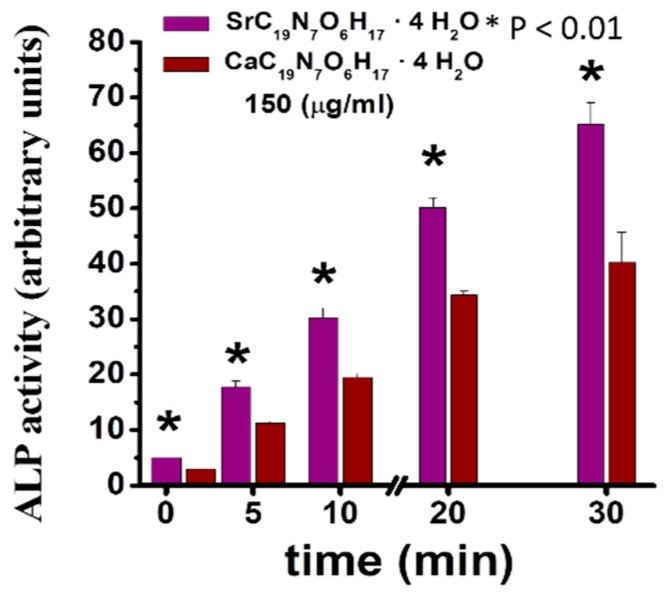
Alkaline phosphatase (ALP) expression in human osteoblasts cultured in the presence of Sr and Ca folic acid derivatives. Figure adapted from Rojo et al. [18] with permission from the Royal society for Chemistry.

**Figure 4:**
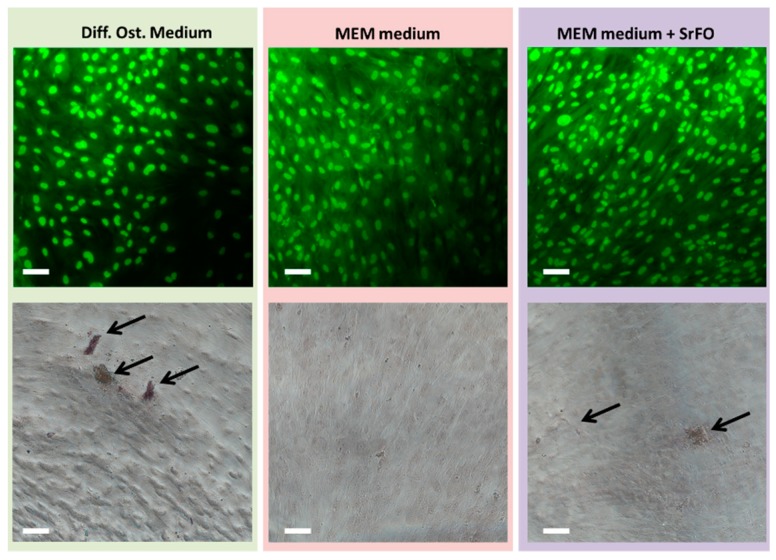
Acridine orange and propidium iodide (OA/PI) (upper) alizarin red (lower) staining of HMSC cultured for four days in osteogenic differentiation medium (left), MEM medium (center) and MEM + Strontium Folate (SrFO) (left). Arrows indicate deposits of calcified extracellular matrix. Scale bar = 100 µm [105].

Sr folate (SrFO) shows low toxicity in vitro in human osteoblasts cultures and human mesenchymal Stem Cells, while inducing a significant expression of alkaline phosphatase (ALP) when compared to its Ca analogue (Figure 3). Studies suggest that SrFO could certainly display important therapeutic properties. Nonetheless, detailed analysis in SrFO physicochemical properties as well as the biological interaction with osteoblast and human stem cells have been conducted (Figure 4).

These studies would allow to state confidently its effect in bone regeneration, and consequently, its potential application in medical therapies, e.g., in chronic-degenerative bone diseases. Bioassays of SrFO and other chosen materials included in ceramic composites has been the subject of our laboratory, with the aim to develop a superior osteogenic scaffold behaving in a dual way, i.e., remodeling bone tissue by stimulating osteogenesis (cell proliferation) from dental pulp and by inhibiting bone resorption induced by osteolysis [18,105].

## 7. SrFO Loaded Biohybrid Scaffold Seeded with Dental Pulp Stem Cells as a New Composite System 

### 7.1. Scaffolds Preparation Procedures and Characterization Techniques

**Figure 5:**
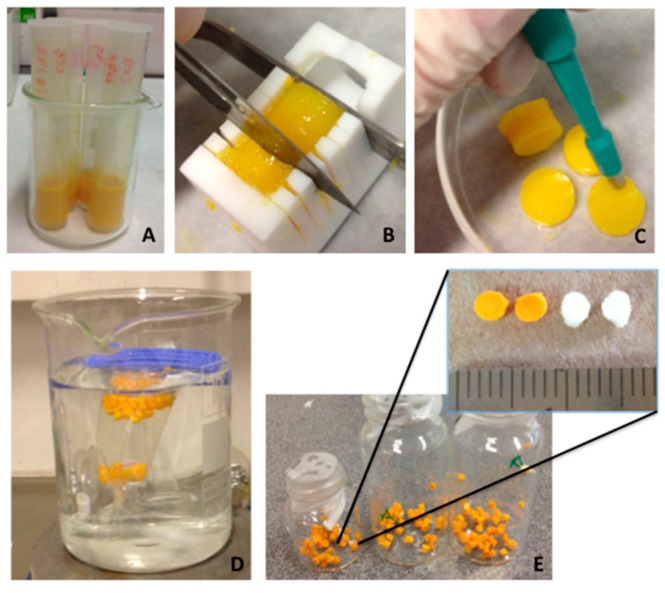
Procedure for scaffold construction as reported in Martin-del-Campo et al. (**A**) Solubilization and mixture of chemical components, (**B**) cutting to slices, (**C**) cutting with punch to obtain discs, (**D**) dialysis with membranes, and (**E**) final product (scaffold) [16].

Increasing interest exist in biodegradable ceramic scaffolds for bone tissue engineering with the ability to deliver active molecules favoring bone formation. Denry et al. showed the action in vivo of strontium-containing glass-ceramic scaffolds, where Sr was successfully incorporated in the newly formed bone, thus increasing significantly the mineral apposition rate evidencing the benefits of in situ release of Sr [10]. Similarly, Tian et al. demonstrated the biocompatibility, osteogenesis (in vivo), and degradability of porous Sr-doped Ca polyphosphate scaffolds in bone substitute applications. Yang et al. [44] synthesized collagen-Sr-HA scaffold to evaluate the in vivo effects of Sr on bone formation [44]. Sr based systems are usually designed to serve as models for the manufacture of scaffolds. To date, only two studies do exist, which are the watersheds in the development of graft substitutes. Recently, Xu et al. tested titanium implants covered with Sr ions chelated with folic acid. The SrFO derivative was stable for long time, while minimal Sr ions release to body fluids. This device promoted the in vitro osteogenic differentiation of MSCs upon implantation. More recently, Martín-del-Campo et al. [16] prepared bioactive porous scaffolds by free radical polymerization of polyethylene glycol methacrylate in the presence of β-tricalcium phosphate (TCP) and SrFO as bioactive compounds (Figure 5) [16]. Roy and Bose tested in vitro the effects of Sr doping in β-TCP on differentiation of mononuclear cells into osteoclast-like cells and its resorptive activity, osteoclast-like cell formation, adhesion and resorption. Hence, substrate chemistry may control osteoclast differentiation and resorptive activity. Furthermore, it may be used for the design of TCP-based resorbable bone substitutes with controlled degradation and osteoclast-like cell formation activity [106].

**Figure 6:**
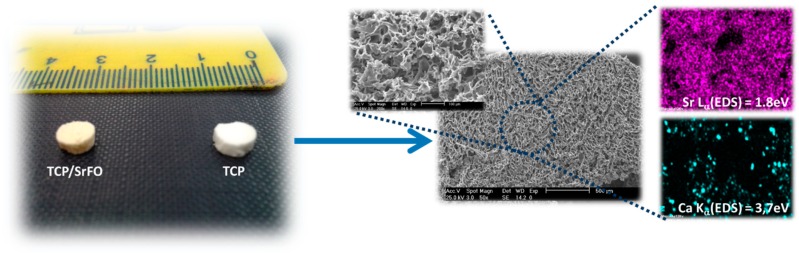
β-tricalcium phosphate (TCP) and TCP/SrFO porous scaffolds obtained by free radical polymerization and lyophilization and surface analysis by scanning electron microscope (SEM) and energy-dispersive X-ray spectroscopy (EDS) spectroscopy [107].

Physical properties, morphology, distribution, size and interconnectivity of pores, and identification of component materials (mapping of elements) were studied in the SrFO loaded biohybrid scaffold by Martín-del-Campo et al. and by San Román et al. (Figure 6) [16,107]. The scaffolds are typically porous with a homogeneous structure and consistent cylindrical shape. Pores are homogeneously distributed displaying interconnected macropores (>100 μm) and micropores (<10 μm) [16]. The above are in agreement with previous studies, thus demonstrating that the presence of Sr in 3D biohybrid scaffolds does not influence the macro- and meso-porous structure in scaffolds. In addition, they provide proper dissolutions rates for Ca and Sr ions and exhibit an excellent microenvironment for cell viability, differentiation and osteogenic activity [19,108,109]. The mapping of elements in scaffolds were performed by energy-dispersive X-ray spectroscopy (EDS) in a scanning electron microscope (SEM) in order to determine the presence of TCP particles and SrFO (Ca and Sr elements respectively) and its dispersion in the matrix.

**Figure 7:**
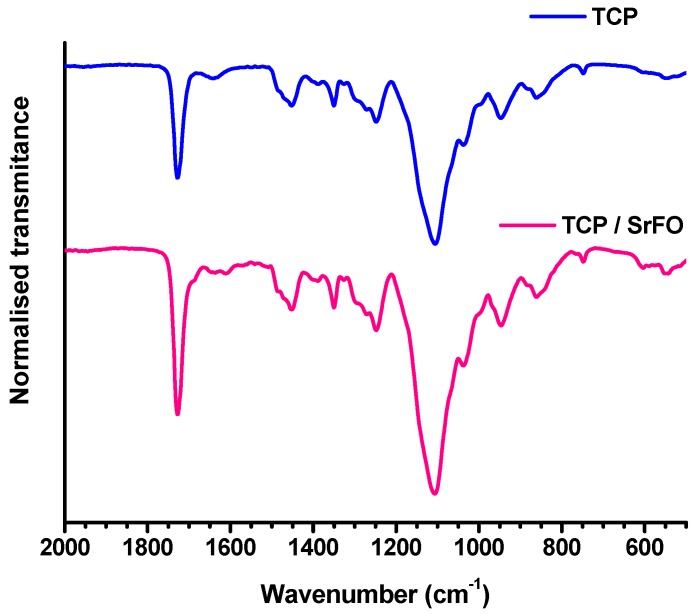
Surface analysis by FTIR-ATR spectroscopy of TCP and TCP/Sr scaffolds. Figure adapted from Martín-del-Campo et al. [16] with permission from the Royal society for Chemistry.

The success of polymerization reaction, and absence of free monomers in TCP and TCP/Sr scaffolds were confirmed by Fourier transform infrared spectroscopy (ATR-FTIR) by the absence of the characteristic vinyl bands (ν) as C=C at 1637 cm^−1^. The presence of a strong vibrating band at 1726 cm^−1^ corresponding to the carbonyl ester of the ceramic component was also discriminated from bands associated to phosphate groups (high and low intensity) observed in the region of 1000 to 500 cm^−1^ (Figure 7) [18].

### 7.2. DPSC Viability on Scaffolds

**Figure 8:**
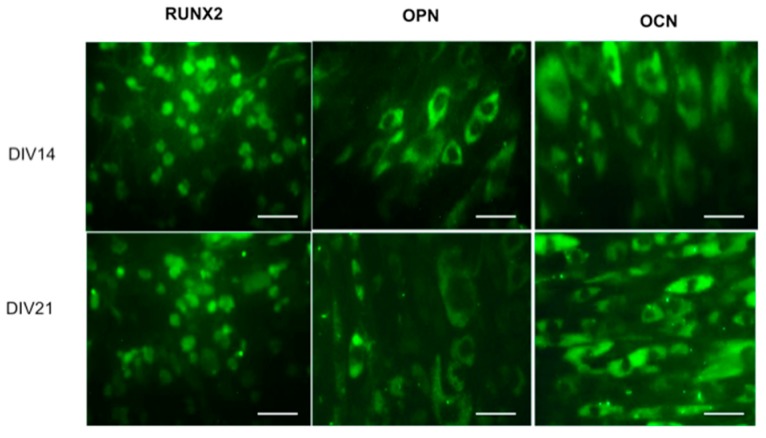
Differentiation of HDPSCs. Expression of RUNX-2, OPN, and OCN at days 14 and 21, (40× magnification). Scale bar 100 μm. Reproduced from Martín-del-Campo et al. [16], with permission from The Royal Society of Chemistry.

Studies suggest that incorporation of Sr ions in bone substitutes constitutes a safe and effective way to stimulate proliferation and differentiation of bone MSCs, osteoblasts, and periodontal ligament cells in vivo [19,20,21]. Recently, Martín-del-Campo et al. demonstrated that incorporation of SrFO into bio-hybrid scaffolds enhances the osteogenic differentiation of HDPSCs. That is, by performing cell culture and characterization of DPSCs, the HDPSCs phenotype and osteogenic profile were confirmed by immunoassays and the expression level of three osteogenic markers (RUNX-2, osteopontin, and osteocalcin), representative of middle and late stages of osteogenic differentiation, were determined histochemically (Figure 8) [16].

**Figure 9:**
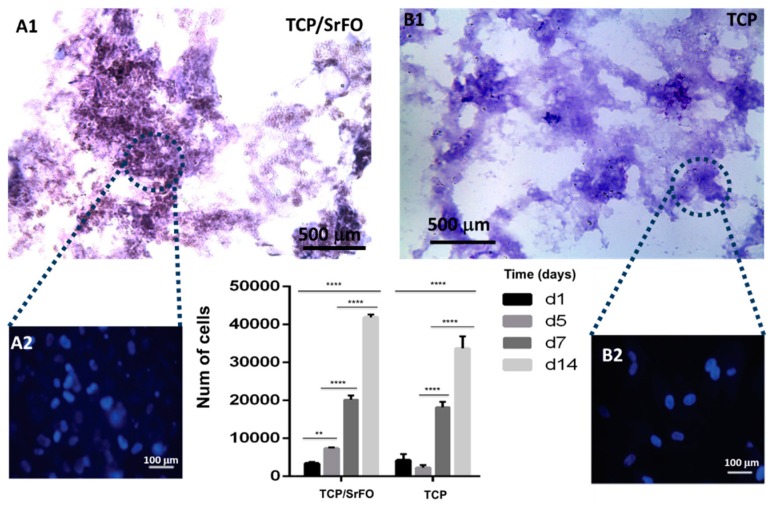
H & E staining on sliced TCP/SrFO (A1) and TCP (B1) scaffolds and nuclei staining (A2 and B2 respectively) of HDPSCs used for the viability quantification. Figure adapted from Martín-del-Campo et al. [16] with permission from The Royal Society of Chemistry. Stars show significant differences throughout days * (*p* < 0.0049) *** (*p* < 0.0001) **** (*p* < 0.0001).

Cell viability and proliferation in composite scaffolds were confirmed, showing a large increase in cell population in TCP/SrFO group. After day seven, H & E viability assay showed a higher cell number in TCP/SrFO scaffolds than the TCP group (Figure 9). Xu et al. [17] confirmed that the high stability of Sr substrates improves cell proliferation in a long term resulting in a remarkably effect on viability of MSCs [17].

### 7.3. Rat Calvarial Defect Model and Implantation of the Scaffold

**Figure 10:**
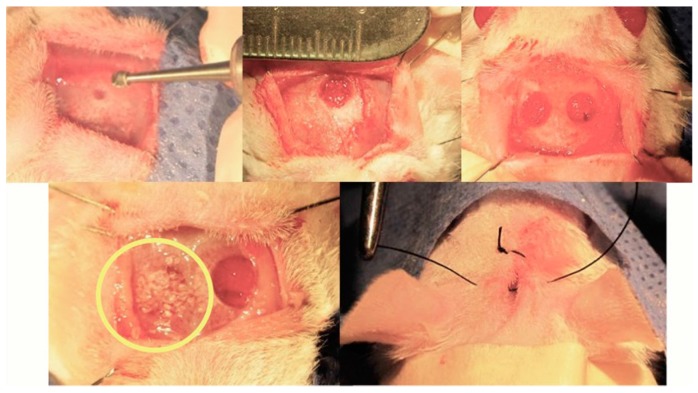
Implementation of the cranial defect model and construct (circle) transplantation. Process reported in Martín-del-Campo et al. [16].

Martín-del Campo et al. implemented a calvarial defect biomodel to perform the implantation of SrFO/TCP scaffolds by filling completely the defective area (Figure 10).

**Figure 11:**
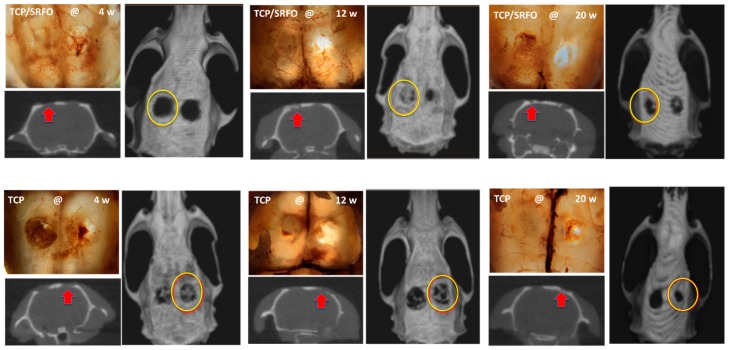
Healing evolution of treated cranial defects mediated by SrFO/TCP (upper) and TCP (lower) composite implants (circle or arrow) and without construct (control). Figure adapted from Martín-del-Campo et al. [16] with permission from The Royal Society of Chemistry.

The biomodels, after implantation of the constructs, showed good recovery and no signs of infection or inflammation as determined by Gross photographic examination and CT analysis. The implanted scaffolds resorb because of formation of newly mineralized tissue. Moreover, radiographic scans of ex vivo bone explant may evidence qualitatively and quantitatively the mineralized tissue in calvaria defects over time. The progressive healing (filling) of cavities is observed in both groups, which are characterized by mineralized tissue and moderate rates in bone growth. Remarkably, the density of the newly bone formed is higher in SrFO group compared with that in TCP (Figure 11) suggesting that Sr promoted the formation of new bone. Yang et al. [44] showed that in HA and Sr-containing scaffolds, the Ca phosphate based scaffold induces the formation of only some mature collagen fibers at the edges of the bone defect, while Sr-containing scaffold induces the formation of mature and remodeled bone at the boundary and in the central bone defect resulting in a relatively high bone density [44].

H & E staining of implants in the bio-model evidenced the formation of mature bone tissue in Sr-systems, displaying a highly organized laminar tissue and the absence of scaffolds remnants. Alizarin red stain when assayed at specific time intervals post-implantation displays the stages of mineralization of the newly formed tissue. In TCP/SrFO scaffolds implants a line like structures is observed indicating the start of mineralization and Ca organization, while osteocytes were observed in matrices indicating on site calcification. Von Kossa stain assay showed the start in calcification, after week 20th, the TCP/SrFO group shows the presence of organized mineralized tissue with the appearance of complete and mature bone. Mineralization is observed through cells and lamellar Ca deposits [16]. Xu et al. confirmed that Sr folic acid derivate may significantly enhance the ALP activity of MSCs in a short-time, and sustain an upregulated ALP activity for longer time. The mineralization of MSCs, as a late stage marker in osteogenic differentiation, demonstrates that Sr folic acid substrates promote MSCs osteogenic differentiation [17].

## 8. Perspectives and Conclusions

SrFO based-scaffolds increase bone regeneration in vivo. The Sr-based-systems seem to be a useful alternative for the regeneration of bone tissue in complicated defects. Seeding with pluripotent stem cells increases its efficacy. This review provides an overview of the known knowledge for the design and manufacture of bio-hybrid scaffolds in tissue engineering combining the benefits of non-protein based morphogens Sr and B vitamins (folates), in combination with stem cells from dental pulp. Pluripotent cells from dental pulp could be considered an excellent alternative as a source of stem cells. Based on published experimental results, it is concluded that HDPSC seeded in hybrid scaffolds stimulates cell growth and differentiation. Furthermore, composite scaffolds containing SrFO are biocompatible and provide an excellent system in the regeneration of bone tissue. SrFO scaffold may be considered an option for the controlled release of Sr and Ca-phosphate. The device, when seeded with HDPSC, offers a potential alternative in the regeneration of bone tissue.
